# Impulsive responding increases during a laboratory model of a cocaine binge in individuals who use cocaine: A preliminary study

**DOI:** 10.46439/addiction.3.010

**Published:** 2025

**Authors:** Nehal P. Vadhan, Sean P. Madden, Majnu John, Stephanie Collins Reed, Suzanne K. Vosburg, John G. Keilp, Richard W. Foltin

**Affiliations:** 1 Northwell, New Hyde Park, NY, USA; 2 Department of Psychiatry, Zucker School of Medicine at Hofstra University, Hempstead, NY, USA; 3 Institute of Behavioral Science, Feinstein Institutes for Medical Research, Manhasset, NY, USA; 4 Department of Psychology, Hofstra University, Hempstead, NY, USA; 5 Department of Psychiatry, New York State Psychiatric Institute, Columbia University Irving Medical Center, New York, NY 10032, USA

**Keywords:** Cocaine, Stimulant, Self-administration, Experimenter-administration, Cognition, Performance, Impulsivity

## Abstract

**Background:**

Individuals with a binge pattern of cocaine use have been found to exhibit cognitive decrements relative to controls, but also experience increases in cognitive performance during binge cocaine administration in the laboratory.

**Objective:**

We examined cognitive performance during binge cocaine administration in the laboratory, but with varied amounts of cocaine, to better estimate its cognitive effects in the natural ecology.

**Methods:**

This study was approved by the Institutional Review Board of the New York State Psychiatric Institute, and all participants provided written informed consent to participate. Twelve individuals who used cocaine regularly (males=91.7%) and were physically/psychiatrically – healthy, completed a nightly counterbalanced cognitive battery during 2 phases of cocaine administration, separated by a period of cocaine abstinence (all phases maximum 5 days each). During cocaine phases, participants smoked cocaine up to 12 times per day (25 mg per occasion) as part of an experimenter-administered or self-administered protocol.

**Results:**

On average, participants exhibited decreased attentional response inhibition (*F*(2,48)=12.70, *p*<0.05) during Binge 1 (M=1072.5 mg cocaine), relative to Abstinence (0.0 mg cocaine) and Binge 2 (M=650.0 mg cocaine). The self-administration group exhibited decreased motor tracking (*F*=6.11(1,12.4), *p*<0.05) during Binge 2, relative to the other study phases, whereas the experimenter-administration group did not.

**Conclusions:**

These data suggest that response inhibition, but not immediate memory or psychomotor speed, was impaired during periods of binge cocaine administration in experienced users. This finding has important implications for behaviors that require inhibitory control, such as driving, during cocaine intoxication.

## Introduction

Individuals who use cocaine exhibit mildly to moderately decreased cognitive performance when cocaine-free for days to months [1,2] relative to controls. However, some studies have not found these group differences [3,4]. This apparent discrepancy could be investigated by examining cocaine use severity, with bingeing patterns (i.e., taking multiple consecutive doses) thought to have greater neuropsychological sequelae than single or well-spaced use [5,6]. In naturalistic studies, greater weekly frequency and total cocaine intake have been associated with cognitive decrements, including deficits in attention and verbal learning [5,7–9]. Longitudinal studies have found that greater reported cocaine use and increasing hair cocaine metabolite levels were associated with concomitant decreases in verbal learning and visual working memory, in a mixed sample of binge and non-binge users [10]. It generally appears that the greater the naturalistic cocaine intake, the greater the cognitive disruption [5].

In contrast, controlled laboratory studies involving individuals with similar experience with cocaine have largely found increased cognitive performance following acute cocaine administration. Single doses of cocaine have enhanced attentional and memory performance [11–13], like other stimulants such as d-amphetamine (e.g., [14]). Longitudinal laboratory studies [15,16] have found enhanced performance on a variety of measures including digit vigilance reaction time and accuracy (assessed daily) during 2 daily “binges” relative to abstinence.

This evidence suggests that binge cocaine use is associated with decreased cognitive performance (measured via clinical tests during non-intoxication) when taken naturalistically, but with enhanced cognitive performance (measured daily with randomized/alternate forms of experimental tests after recent cocaine use) when taken in laboratory settings. The distinctive methodologies may play a role in these discrepant findings. Thus, to better approximate the natural ecology, we examined data from binge phases with variation in the amount of cocaine administered, since cocaine use amount varies within naturalistic binges, and counterbalanced cognitive task order, since cognitive demands in the natural ecology are unlikely to be in a fixed order. We hypothesized that cognitive performance would be: 1) better during the binge phases compared to the abstinence phase, 2) inversely associated with the amount of cocaine taken during the binge phases, and 3) better for healthy controls than the participants who use cocaine.

## Materials and Methods

This study was approved by the Institutional Review Board of the New York State Psychiatric Institute (approvals 4242 and 4529) and was carried out following the rules of the Declaration of Helsinki of 1975 [17]. All participants provided written informed consent and were compensated for their time. The submitted manuscript adheres to the ICMJE Recommendations [18]. All data were collected at Columbia University Irving Medical Center.

### Participants

Twelve physically/psychiatrically healthy, nontreatment-seeking, individuals who reported using cocaine regularly and ranged in age from 29–44 (mean=39.2 years; SD=4.8), were predominantly male (11/12; 91.7%) participated in this study. Participants must have reported smoking cocaine at least twice per week for the previous 6 months and tested positive for cocaine metabolites on screening urine toxicology tests to be eligible. On average, participants reported using cocaine 4.3 (SD=1.9) days per week and spending on average approximately $346 (SD=304.3) on cocaine weekly (approximately 3.54 grams [SD=3.11], as estimated by cocaine purchase data (U.S. Drug Enforcement Administration, 2008)).

Each participant was enrolled in 1 of 2 within-participant laboratory studies (described below) where cocaine was either delivered in an experimenter-administered (n=7) or a self-administered (n=5) protocol. These groups did not differ (p>0.05) on age, sex, or reported cocaine use patterns.

Task data from 19 healthy controls (with no lifetime history of cocaine use or psychiatric illness) were included as a reference point for normative cognitive performance. Controls were comparable in age (range=28–56; mean=39.58; SD=7.84) and were also predominantly male (15/19; 78.9%). They completed the same tasks a single time in a controlled laboratory setting with no drug administration, as part of a broader study (i.e., [19]) on neuropsychological functioning in substance users.

### Experimental design

Cocaine base, derived from cocaine hydrochloride, was prepared in pellets of 25 mg by the New York State Psychiatric Institute Pharmacy, as previously described [20,21].

In the experimenter-administration protocol [20], participants were instructed to smoke 25 mg of cocaine at 14 min intervals, 6 times per session (2 sessions per day: 9:00 am and 3:00 pm). Cocaine sessions were separated by brief phases of monitored abstinence. There were 4 days of cocaine sessions and 3 days of abstinence during each stage of the study (2 phases in total), and 1 day of abstinence between each phase. During each cocaine phase, participants smoked up to 48 doses of 25 mg cocaine (1,200 mg).

In the self-administration protocol [21], participants had 6 opportunities each session to smoke 25 mg of cocaine or choose to receive a probabilistic monetary reinforcer ($0.00–120.00 per choice). Choices occurred at 14 min intervals and 6 times per session (2 sessions per day: 9:30 am and 1:30 pm). The first phase of the study (binge 1) consisted of 5 consecutive days of cocaine sessions, where each participant had an opportunity to take up to 12–25 mg doses (300 mg) per day (total of 60 doses of cocaine; 1,500 mg); the second phase included 9 days of enforced abstinence (0 doses of cocaine), and the last phase consisted of 2 days of cocaine sessions (binge 2), with an opportunity to take up to 12–25 mg doses (300 mg) each day for a total of 24 doses (600 mg). Individual data on cardiovascular and subjective states were no longer available for analysis, but cocaine administered in these fashions has been reliably shown to elicit significant increases in these states [20,22].

Cognitive data were available for up to 14 days of participation (see Table 1 for daily participant sample sizes). Aggregating participants’ cognitive data across both studies, 3 study phases across 14 days were identified for which cognitive data were taken: 1) Binge 1 (4–5 days duration), 2) Abstinence (4–5 days duration), and 3) Binge 2 (2–4 days duration).

### Cognitive assessment

For the cocaine group, cognitive performance was assessed approximately 30 min following the last scheduled dose (or its equivalent time for Abstinence days), for up to 5 days per study phase (i.e., Binge 1, Abstinence, and Binge 2) and for up to 14 total sessions. Three computerized/repeatable tasks (described below) were administered with task order counterbalanced, and stimulus presentation randomized. To familiarize participants with the tasks 1–2 training sessions were administered prior to the study.

The Digit Symbol Substitution Test (DSST; [23] assessed psychomotor function through a task in which geometric stimuli associated with different numbers were reproduced under timed conditions. Total percent correct was examined.

The Digit-Recall Task (DRT; [24] assessed immediate visual memory through a procedure in which numeric stimuli (i.e., 8-digit number strings) were reproduced both during and after they appeared on the computer screen. Total number of correctly copied stimuli (before recall) and percent correctly reproduced (immediate recall) were examined.

The Divided Attention Task (DAT; [25] assessed divided attention and impulsivity through a task in which participants tracked a moving circle that increased in speed (when tracked accurately), while simultaneously responding to target stimuli (i.e., small black square appearing on corner of screen). Average number of false alarms (i.e., responses made when the black square was not on the screen) and maximal circle speed were examined.

### Statistical analyses

Performance for each cognitive task was analyzed as a function of cocaine phase alone (binge 1 vs abstinence vs binge 2) and by day (day 1 vs 2, day 1 vs 3, etc). The number of cocaine doses taken during each cocaine phase was compared with paired-sample t-tests. Post-hoc pairwise comparisons probed any significant main effects of phase. Significant phase × day interactions were probed in a hierarchical fashion. First, tests of simple main effects were used to confirm whether the trajectory of cognitive performance (within each phase) was significantly different from a flat line. Phases with significant simple main effects were then further probed with post-hoc pairwise comparisons to uncover any significant differences between phase days.

Missing data was assumed to be missing at random; thus, analysis of the data was conducted by using a mixed-models approach, which accounts for the within subject correlation of repeated measurements. *P* values and *F*- or *T*-statistics for the main effects of cocaine phase (binge 1, abstinence, and binge 2) and day, as well as cocaine phase-by-day interaction were based on the type 3 tests of fixed effects. In the type 3 tests for fixed effects, the denominator degrees of freedom for *F*-statistic were computed in accordance with Satterthwaite’s formula, which is robust against variance heterogeneity and considers the variability within the group and the sample size. Analyses were performed using the PROC MIXED procedure in SAS 9.4., with α=0.05 for all measures. Tests of simple main effects were conducted using the “slice” option available in the ‘LS Means’ statement within PROC MIXED.

Given that self-administered cocaine may produce differing physiological effects than experimenter-administered cocaine [26–28], we further explored differences in cognitive performance as a function of study protocol group (experimenter- vs self-administered), cocaine phase (binge 1 vs abstinence vs binge 2) and total cocaine doses taken (covariate) with analogous analyses. As a secondary correction for decreasing sample size over time, we performed all of the above analyses on a dataset restricted to the 1^st^ two days of each study phase. Finally, we compared cognitive performance between individuals who use cocaine and healthy controls, with independent samples t-tests. We utilized the data from the first day of the abstinence phase (i.e., Day 6), as it was judged to be most comparable to the controls’ data (single task administration).

## Results

### Cocaine doses

More cocaine was taken during Binge 1 (M=42.9 doses (1,072.5 mg), SD=14.9 (372.5 mg) relative to Binge 2 (M=26.0 doses (650 mg), SD=19.4 (485 mg); (*t*(9)=2.7, *p*=0.02).

### Cognitive performance by task

#### DSST:

There was no main effect of cocaine phase (*F* 0.53, *df*=2,47, *p*=0.59), nor an interaction between cocaine phase and day (*F* =0.96, *df*=8,83.7, *p*=0.48) on total percent correct.

#### DRT:

There was no main effect of cocaine phase (*F*=2.98, *df*=2,50.9, *p*=0.06), nor a cocaine phase × day interaction (*F*=1.19, *df*=8,90.6, *p*=0.32) on number correct. On percent immediate recall, there was no main effect of cocaine phase (*F*=1.38, *df*=2,50.1, *p*=0.26), nor a cocaine phase × day interaction (*F*=0.31, *df*=8,84.5, *p*=0.96).

#### DAT:

There was a significant main effect of cocaine phase on number of false alarms (*F*=12.70, *df*=2,48.6, *p*=0.002), but no cocaine phase × day interaction (*F*=0.83, *df*=8,96.3, *p*=0.58). More false alarms (2.86–3.33 times as many) occurred during Binge 1 relative to Abstinence (*t*(44.8)=4.56, *p*<0.001) and Binge 2 (*t*(47)=3.92, *p*<0.0003). Thus, attentional response inhibition was relatively impaired during the 1^st^ cocaine binge. There was no main effect of cocaine phase (*F*=1.13, *df*=2,45.2, *p*=0.33) nor a significant cocaine phase × day interaction (*F*=1.97, *df*=8,84.2, *p*=0.06) on maximal speed.

Directions and patterns of significance remained the same for all analyses when only considering the 1^st^ two days of each binge phase (See Table S1).

### Protocol type subgroup analyses

On DAT maximal speed (with total cocaine doses covaried), there was a main effect of protocol type (*F*=6.11, *df*=1,12.4, *p*=0.03), with the self-administration group exhibiting decreased performance overall relative to the experimenter-administration group, (*t*(12.4)=−2.47, *p*=0.03). There was also a protocol type × cocaine phase interaction (*F*=10.87, *df*=2,49, *p*<0.001). The test of simple main effects was significant for Binge 2 (*F*=13.20, *df*=1, 16.6, *p*<0.01), and within Binge 2, the self-administration group exhibited reduced maximal speed relative to the experimenter-administration group (*t*(16.6)=−3.63, *p*<0.01). No other differences as a function of protocol type were found (*p*’s>0.05), and directions and patterns of significance were similar when only considering the 1^st^ two days of each binge phase.

### Comparisons between participants who use cocaine and controls

Controls exhibited better performance on DSST percent correct (*t*(10.6)=2.55, *p*=0.03) and DAT maximal speed (*t*(13.9)=3.44, *p*<0.01), relative to participants who use cocaine. The groups did not differ significantly on any other measures (*p’s*>0.05).

## Discussion

In this aggregate analysis of 2 human laboratory studies, it was found that psychomotor function (DSST), digit recall (DRT), and motor tracking (DAT) did not change between alternating binge cocaine administration and abstinence. However, complex attentional response inhibition (DAT # false alarms) was decreased during the first multi-day smoked cocaine binge, relative to enforced abstinence and a 2^nd^ cocaine binge (where relatively less cocaine was taken). Finally, the performance of participants who use cocaine was decreased relative to healthy controls psychomotor function and motor tracking at a single timepoint.

Thus, our 1^st^ hypothesis that performance would be better during cocaine use relative to abstinence was not supported. Our 2^nd^ hypothesis that performance would be inversely associated with the amount of cocaine use was partially supported in that DAT performance was decreased during a higher intensity cocaine binge (M=1,072.5 mg) relative to a lower intensity cocaine binge (M=650 mg). Our 3^rd^ hypothesis that performance would be better in the healthy control group was supported.

However, our findings are inconsistent with prior laboratory studies, which have shown enhanced cognitive performance during periods of cocaine use, relative to periods of abstinence [1,15]. They may be more consistent with prior naturalistic studies, which have shown an inverse relationship between cocaine use severity and cognitive performance (e.g., [5,10]), and increased performance- and self-report - based impulsivity, in individuals who use stimulants compared to controls [29–31]. Given the similarities in participant characteristics and longitudinal study design under controlled conditions to the other laboratory studies, it is possible that our altered methodology to increase ecological validity (e.g., variation in cocaine amount, counterbalanced task order) may have contributed to this apparent discrepancy. Finally, the participants with cocaine use exhibited performance during a time of abstinence that was overall lower than or equivalent to the control reference data, broadly consistent with the literature.

Exploratory analyses indicated one difference in cognitive performance as a function of cocaine administration protocol, with the experimenter-administration group attaining greater maximal speed on a divided attention task than the self-administration group (accounting for total cocaine doses taken). It is possible that the increased physiologically-stimulating effects of experimenter-administered cocaine (relative to self-administration; [26]) led to this difference, although unmeasured pre-existing group differences may also have played a role.

This study’s limitations (shared in part with previous studies; e.g., [16,26] included its small sample size, non-counterbalanced drug conditions and lack of placebo control, which were partially mitigated by its ABA design, counterbalanced cognitive task order, and confirmatory analyses restricted to days with minimal missing data. Data were also pooled across two different studies, but this enabled us to optimize our data inclusion and examine cocaine administration methodology as an independent factor. Finally, our healthy control data were only collected at a single time point, prohibiting comparisons to the experimental group’s longitudinal data. Notwithstanding these limitations, our findings may lend support to the notion that use of relatively greater amounts of cocaine is associated with decreased response inhibition, while use of relatively moderate amounts of cocaine is not. Future studies should address these limitations for optimal investigation of the interaction between binge cocaine use, cognitive performance and time. This is an important endeavor, since cognitive function is a well-documented and -characterized predictor of driving behavior [32] and treatment outcomes for cocaine use disorder [33].

## Conclusion

In this human laboratory study, we found that attentional response inhibition was impaired during a high-intensity smoked cocaine binge but not during a period of abstinence or a lower-intensity binge. While these data are preliminary, they suggest that greater cocaine intake is selectively associated with decreased inhibitory control, diverging from prior laboratory studies. Further research is needed to explore the multifactorial interactions between cocaine use and cognition.

## Supplementary Material

ASA-25-010-Supplementary file

## Figures and Tables

**Table 1.** Cognitive performance data: number and percentage of participants contributing data by task and day, and mean performance per day and study phase.

Study phase:			Binge 1					Abstinence					Binge 2			
Task	Control reference data (M (SE))		Day 1	Day 2	Day 3	Day 4	Day 5	Day 6	Day 7	Day 8	Day 9	Day 10	Day 11	Day 12	Day 13	Day 14
DSST		Daily n (%)	11 (96.7)	11 (96.7)	11 (96.7)	9 (75.0)	4 (33.3)	11 (96.7)	11 (96.7)	10 (83.3)	10 (83.3)	3 (25.0)	9 (75.0)	8 (66.7)	3 (25.0)	2 (16.7)
% correct	0.97 (0.01)	Daily M (SE)	0.90 (0.04)	0.87 (0.04)	0.87 (0.04)	0.91 (0.04)	0.85 (0.05)	0.90 (0.04)	0.89 (0.04)	0.95 (0.04)	0.92 (0.04)	0.77 (0.05)	0.90 (0.04)	0.90 (0.04)	0.89 (0.07)	0.87 (0.09)
		Phase M (SE)	0.89 (0.03)					0.89 (0.03)					0.91 (0.03)			
DRT		Daily n (%)	11 (96.7)	11 (96.7)	10 (83.3)	8 (66.7)	4 (33.3)	11 (96.7)	11 (96.7)	10 (83.3)	10 (83.3)	4 (33.3)	10 (83.3)	8 (66.7)	3 (25.0)	2 (16.7)
# correct	6.84 (0.37)	Daily M (SE)	6.99 (0.42)	6.63 (0.42)	6.50 (0.43)	6.78 (0.44)	6.77 (0.51)	6.85 (0.43)	6.39 (0.43)	7.13 (0.43)	6.84 (0.44)	7.33 (0.51)	7.07 (0.40)	7.36 (0.42)	7.03 (0.56)	7.10 (0.64)
		Phase M (SE)	6.74 (0.35)					6.81 (0.35)					7.18 (0.36)			
Immediate recall	0.69 (0.07)	Daily M (SE)	0.64 (0.09)	0.64 (0.09)	0.57 (0.10)	0.67 (0.10)	0.64 (0.12)	0.46 (0.10)	0.55 (0.10)	0.54 (0.10)	0.51 (0.10)	0.54 (0.12)	0.62 (0.09)	0.67 (0.10)	0.58 (0.16)	0.75 (0.21)
		Phase M (SE)	0.59 (0.08)					0.56 (0.08)					0.63 (0.08)			
DAT		Daily n (%)	10 (83.3)	11 (96.7)	11 (96.7)	9 (75.0)	4 (33.3)	11 (96.7)	11 (96.7)	11 (96.7)	10 (83.3)	4 (33.3)	10 (83.3)	8 (66.7)	3 (25.0)	2 (16.7)
# false alarms	0.25 (0.08)	Daily M (SE)	0.23 (0.05)	0.14 (0.05)	0.13 (0.05)	0.23 (0.05)	0.11 (0.07)	0.10 (0.05)	0.07 (0.05)	0.09 (0.05)	0.15 (0.05)	0.09 (0.07)	0.04 (0.05)	0.03 (0.05)	0.04 (0.08)	0.01 (0.10)
		Phase M (SE)	**0.20 (0.03)** *					0.07 (0.03)					0.06 (0.03)			
Maximum speed	4.55 (0.17)	Daily M (SE)	2.97 (0.38)	3.51 (0.38)	2.79 (0.38)	3.07 (0.39)	3.18 (0.45)	3.29 (0.39)	3.05 (0.39)	3.21 (0.39)	2.79 (0.45)	3.04 (0.36)	3.09 (0.38)	3.90 (0.50)	3.61 (0.57)	4.37 (0.73)
		Phase M (SE)	3.01 (0.33)					3.24 (0.33)					3.18 (0.34)			

DSST: Digit-Symbol Substitution Test; DRT: Digit-Recall Task; DAT: Divided Attention Task;

* *p*<0.05, Binge 1 differed significantly from Abstinence and Binge 2.

## References

[1] Spronk DB, van Wel JH, Ramaekers JG, Verkes RJ. Characterizing the cognitive effects of cocaine: a comprehensive review. Neurosci Biobehav Rev. 2013 Sep;37(8):1838–59. 23876288 10.1016/j.neubiorev.2013.07.003

[2] Vonmoos M, Hulka LM, Preller KH, Jenni D, Baumgartner MR, Stohler R, e al. Cognitive dysfunctions in recreational and dependent cocaine users: role of attention-deficit hyperactivity disorder, craving and early age at onset. Br J Psychiatry. 2013 Jul;203(1):35–43. 23703315 10.1192/bjp.bp.112.118091

[3] Frazer KM, Richards Q, Keith DR. The long-term effects of cocaine use on cognitive functioning: A systematic critical review. Behav Brain Res. 2018 Aug 1;348:241–62. 29673580 10.1016/j.bbr.2018.04.005

[4] Jovanovski D, Erb S, Zakzanis KK. Neurocognitive deficits in cocaine users: a quantitative review of the evidence. J Clin Exp Neuropsychol. 2005 Feb;27(2):189–204. 15903150 10.1080/13803390490515694

[5] Almeida PP, de Araujo Filho GM, Malta SM, Laranjeira RR, Marques ACRP, Bressan RA, Attention and memory deficits in crack-cocaine users persist over four weeks of abstinence. J Subst Abuse Treat. 2017 Oct;81:73–8. 28847458 10.1016/j.jsat.2017.08.002

[6] Potvin S, Stavro K, Rizkallah E, Pelletier J. Cocaine and cognition: a systematic quantitative review. J Addict Med. 2014 Sep-Oct;8(5):368–76. 25187977 10.1097/ADM.0000000000000066

[7] Lima DR, Gonçalves PD, Ometto M, Malbergier A, Amaral RA, Dos Santos B, The role of neurocognitive functioning, substance use variables and the DSM-5 severity scale in cocaine relapse: A prospective study. Drug Alcohol Depend. 2019 Apr 1;197:255–61. 30875646 10.1016/j.drugalcdep.2019.01.013

[8] Bolla KI, Rothman R, Cadet JL. Dose-related neurobehavioral effects of chronic cocaine use. J Neuropsychiatry Clin Neurosci. 1999 Summer;11(3):361–9. 10440013 10.1176/jnp.11.3.361

[9] Bolla KI, Funderburk FR, Cadet JL. Differential effects of cocaine and cocaine alcohol on neurocognitive performance. Neurology. 2000 Jun 27;54(12):2285–92. 10881254 10.1212/wnl.54.12.2285

[10] Vonmoos M, Hulka LM, Preller KH, Minder F, Baumgartner MR, Quednow BB. Cognitive impairment in cocaine users is drug-induced but partially reversible: evidence from a longitudinal study. Neuropsychopharmacology. 2014 Aug;39(9):2200–10. 24651468 10.1038/npp.2014.71 PMC4104339

[11] Spronk DB, De Bruijn ER, van Wel JH, Ramaekers JG, Verkes RJ. Acute effects of cocaine and cannabis on response inhibition in humans: an ERP investigation. Addict Biol. 2016 Nov;21(6):1186–98. 26037156 10.1111/adb.12274

[12] Johnson B, Overton D, Wells L, Kenny P, Abramson D, Dhother S, Effects of acute intravenous cocaine on cardiovascular function, human learning, and performance in cocaine addicts. Psychiatry Res. 1998 Jan 16;77(1):35–42. 10710173 10.1016/s0165-1781(97)00127-3

[13] Hutten NR, Kuypers KP, van Wel JH, Theunissen EL, Toennes SW, Verkes RJ, A single dose of cocaine enhances prospective memory performance. J Psychopharmacol. 2018;32(8):883–92. 29947572 10.1177/0269881118783299 PMC6058404

[14] Dolder PC, Strajhar P, Vizeli P, Odermatt A, Liechti ME. Acute effects of lisdexamfetamine and D-amphetamine on social cognition and cognitive performance in a placebo-controlled study in healthy subjects. Psychopharmacology (Berl). 2018;235(5):1389–402. 29511807 10.1007/s00213-018-4849-0

[15] Pace-Schott EF, Stickgold R, Muzur A, Wigren PE, Ward AS, Hart CL, Cognitive performance by humans during a smoked cocaine binge-abstinence cycle. Am J Drug Alcohol Abuse. 2005;31(4):571–91. 16320435 10.1081/ada-200068120

[16] Pace-Schott EF, Morgan PT, Malison RT, Hart CL, Edgar C, Walker M, Cocaine users differ from normals on cognitive tasks which show poorer performance during drug abstinence. Am J Drug Alcohol Abuse. 2008;34(1):109–21. 18161649 10.1080/00952990701764821

[17] Association WM. World Medical Association Declaration of Helsinki: Ethical Principles for Medical Research Involving Human Participants. Jama. 2025;333(1):71–4. 39425955 10.1001/jama.2024.21972

[18] Editors ICoMJ. Recommendations for the Conduct, Reporting, Editing, and Publication of Scholarly Work in Medical Journals. 2025 Available from: https://www.icmje.org/. 25558501

[19] Vadhan NP, Myers CE, Benedict E, Rubin E, Foltin RW, Gluck MA. A decrement in probabilistic category learning in cocaine users after controlling for marijuana and alcohol use. Exp Clin Psychopharmacol. 2014 Feb;22(1):65–74. 24188172 10.1037/a0034506

[20] Reed SC, Haney M, Evans SM, Vadhan NP, Rubin E, Foltin RW. Cardiovascular and subjective effects of repeated smoked cocaine administration in experienced cocaine users. Drug Alcohol Depend. 2009 Jun 1;102(1–3):102–7. 19303723 10.1016/j.drugalcdep.2009.02.004 PMC2679224

[21] Vosburg SK, Haney M, Rubin E, Foltin RW. Using a novel alternative to drug choice in a human laboratory model of a cocaine binge: a game of chance. Drug Alcohol Depend. 2010 Jul 1;110(1–2):144–50. 20346597 10.1016/j.drugalcdep.2010.02.015 PMC2931590

[22] Foltin RW, Haney M. Conditioned effects of environmental stimuli paired with smoked cocaine in humans. Psychopharmacology (Berl). 2000 Mar;149(1):24–33. 10789879 10.1007/s002139900340

[23] McLeod DR, Griffiths RR, Bigelow GE, Yingling J. An automated version of the digit symbol substitution test (DSST). Behavior Research Methods & Instrumentation. 1982 Sep;14(5):463–6.

[24] Hart CL, van Gorp W, Haney M, Foltin RW, Fischman MW. Effects of acute smoked marijuana on complex cognitive performance. Neuropsychopharmacology. 2001 Nov;25(5):757–65. 11682259 10.1016/S0893-133X(01)00273-1

[25] Miller TP, Taylor JL, Tinklenberg JR. A comparison of assessment techniques measuring the effects of methylphenidate, secobarbital, diazepam and diphenhydramine in abstinent alcoholics. Neuropsychobiology. 1988;19(2):90–6. 3226529 10.1159/000118441

[26] Donny EC, Bigelow GE, Walsh SL. Comparing the physiological and subjective effects of self-administered vs yoked cocaine in humans. Psychopharmacology (Berl). 2006 Jul;186(4):544–52. 16552557 10.1007/s00213-006-0312-8

[27] Dworkin SI, Mirkis S, Smith JE. Response-dependent versus response-independent presentation of cocaine: differences in the lethal effects of the drug. Psychopharmacology (Berl). 1995 Feb;117(3):262–6. 7770601 10.1007/BF02246100

[28] Hemby SE, Co C, Koves TR, Smith JE, Dworkin SI. Differences in extracellular dopamine concentrations in the nucleus accumbens during response-dependent and response-independent cocaine administration in the rat. Psychopharmacology (Berl). 1997 Sep;133(1):7–16. 9335075 10.1007/s002130050365 PMC13463260

[29] Foltin RW, Luba R, Chen Y, Wang Y, Evans SM. Impulsivity in cocaine users compared to matched controls: Effects of sex and preferred route of cocaine use. Drug Alcohol Depend. 2021 Sep 1;226:108840. 34246916 10.1016/j.drugalcdep.2021.108840 PMC8355072

[30] Ersche KD, Turton AJ, Chamberlain SR, Müller U, Bullmore ET, Robbins TW. Cognitive dysfunction and anxious-impulsive personality traits are endophenotypes for drug dependence. Am J Psychiatry. 2012 Sep;169(9):926–36. 22952072 10.1176/appi.ajp.2012.11091421 PMC3533378

[31] Kaboodvand N, Shabanpour M, Guterstam J. Neural correlates of impulsivity in amphetamine use disorder. Psychiatry Res Neuroimaging. 2024 Sep;343:111860. 38991286 10.1016/j.pscychresns.2024.111860

[32] Apolinario D, Magaldi RM, Busse AL, Lopes LDC, Kasai JYT, Satomi E. Cognitive impairment and driving: A review of the literature. Dement Neuropsychol. 2009 Oct-Dec;3(4):283–90. 29213641 10.1590/S1980-57642009DN30400004 PMC5619413

[33] Mahoney JJ. Cognitive dysfunction in individuals with cocaine use disorder: Potential moderating factors and pharmacological treatments. Exp Clin Psychopharmacol. 2019 Jun;27(3):203–14. 30556731 10.1037/pha0000245 PMC6538444

