# Supplementary material for: Impulsive responding increases during a laboratory model of a cocaine binge in individuals who use cocaine: A preliminary study
Source: Addict Subst Abus (Middlet). Author manuscript; Available in PMC 2026 Feb 19. (PMC12916013; doi:10.46439/addiction.3.010)
Supplement: ASA-25-010-Supplementary file [file NIHMS2146679-supplement-ASA-25-010-Supplementary_file.pdf]

Citation: Vadhan NP, Madden SP, John M, Reed SC, Vosburg SK, Keilp JG, et al. Impulsive responding increases during a laboratory model of a cocaine binge in individuals who use cocaine: A preliminary study. Addict Subst Abuse. 2025;3(1):12-17.

Table S1. Cognitive Performance During the First Two Days of Each Phase

		Binge 1		Abstinence		Binge 2		Mixed Methods Results				
	Time	D1	D2	D1	D2	D1	D2	n	Main effect of drug condition		Drug condition × time interaction	
**TASK**									Test value	*p*	Test value	*p*
DSST (% correct)	M	91.2	87.0	88.3	91.0	90.4	91.0	12	F(2,34.8) = 0.13	0.88	F(2,39.9) = 1.99	0.15
	SEM	0.0	0.0	0.0	0.0	0.0	0.0					
DRT copy (# correct)	M	7.1	6.6	6.5	6.7	7.0	6.8	12	F(2,26.1) = 0.20	0.82	F(2,40.7) = 1.89	0.16
	SEM	0.4	0.4	0.5	0.5	0.5	0.5					
DRT imm rec. (%)	M	0.7	0.7	0.5	0.5	0.8	0.7	12	F(2,29.8) = 1.55	0.23	F(2,39.2) = 0.84	0.44
	SEM	0.1	0.1	0.1	0.1	0.1	0.1					
**DAT false alarms (#)**	**M**	**0.2**	**0.1**	0.1	0.1	**0.0**	**0.0**	12	**F(2,39.8) = 4.70**	**0.01**	F(2,37.7) = 0.75	0.48
	SEM	0.0	0.0	0.1	0.1	0.1	0.1					
DAT max speed	M	2.7	3.4	3.1	3.2	3.3	3.0	12	F(2,21.3) = 0.02	0.98	F(2,40.2) = 2.42	0.10
	SEM	0.4	0.4	0.5	0.5	0.5	0.5					

Note. DSST, Digit Symbol Substitution Test; DRT imm rec., Digit-Recall Task, immediate recall; DAT, Divided Attention Task; D1, Day 1; D2, Day 2.
